# The Protective Effect of Low Dose of Lipopolysaccharide Pretreatment on Endotoxin-Induced Uveitis in Rats Is Associated with Downregulation of CSF-1 and Upregulation of LRR-1

**DOI:** 10.1155/2020/9314756

**Published:** 2020-07-01

**Authors:** Yunli Ling, Jing Wang, Shuo Yu, Wenjie Li, Hong Lu

**Affiliations:** ^1^Department of Ophthalmology, Beijing Chao-Yang Hospital, Capital Medical University, No. 8 South Gongren Tiyuchang Road, Chaoyang District, Beijing 100020, China; ^2^Department of Ophthalmology, Peking University Third Hospital, Beijing 100083, China; ^3^Department of Ophthalmology, Lanzhou University First Hospital, Lanzhou 730000, China

## Abstract

**Purpose:**

To observe the effect of low dose of lipopolysaccharide (LPS) pretreatment on the expression of CSF-1 and LRR-1 in rats with endotoxin-induced uveitis (EIU), and to explore the possible role of TLR4.

**Method:**

EIU was induced by a single subcutaneous injection of 200 *μ*g LPS. For the endotoxin tolerance group, the induction of EIU was preceded by a daily subcutaneous injection of 0.1 mg/kg LPS for five days. Clinical scores were graded at 24 h after EIU under a slit lamp microscope. HE stain was performed to observe the histopathology. The concentrations of IL-17, INF-*γ*, and IL-6 in aqueous humor were quantified with enzyme-linked immunosorbent assay. Real-time PCR, Western blot, and immunofluorescence analysis were used to determine the expression of NF-*κ*B P65 and the activation of CSF-1, LRR-1.

**Results:**

: Low dose of LPS pretreatment produced a suppressive effect by significantly reducing the inflammatory reaction of anterior segment as measured by slit lamp and histopathology. It also significantly reduced the concentrations of IL-17, INF-*γ*, and IL-6 in aqueous humor and the expression of CSF-1 and NF-*κ*B P65, while increased the expression of LRR-1 compared to the EIU group.

**Conclusions:**

Low dose of LPS pretreatment can ameliorate endotoxin-induced uveitis in rats. This protection may be associated with upregulation of LRR-1 and downregulation of CSF-1, which is regulated by TLR4 signaling pathway.

## 1. Introduction

Uveitis represents a group of conditions characterised by intraocular inflammation. The term uveitis includes iritis, cyclitis, and choroiditis; however, it now encompasses inflammation of adjacent intraocular structures such as the retina, vitreous, and optic nerve [1]. Endotoxin-induced uveitis (EIU) is an efficient animal model to study pathological mechanisms associated with the disease [2]. It is mainly manifested as signs of acute anterior uveitis, such as ciliary congestion, iris blood vessels dilatation, anterior chamber exudate, pupil occlusion, and fibrous membrane formation. The expression of Toll-like receptor (TLR) 4, MyD88, and Nuclear factor-*κ*B (NF-*κ*B) P65 in rats' iris ciliary body tissues change with time and are closely related to the degree of inflammation [3, 4]. The activation of NF-*κ*B P65 mediated by TLR4 signaling pathway is the key of acute anterior uveitis [5].

As a cell wall component of gram-negative bacteria, lipopolysaccharide (LPS) can induce a strong inflammatory response through the TLR4 signaling pathway, with a series of inflammatory factors, like interferon-*γ* (INF-*γ*), interleukin-6 (IL-6), and interleukin-17 (IL-17) releasing. However, a low dose of LPS stimulation cannot induce the stable expression of proinflammatory mediators. The capacity of a cell to respond to LPS challenge is reduced. The body becomes tolerant to subsequent exposure to a lethal dose of LPS and tissue damage caused by inflammatory reaction is significantly reduced; this phenomenon is called endotoxin tolerance (ET) [6]. Accumulating evidences [7, 8] show that a low dose of LPS pretreatment can relieve the intraocular inflammation in EIU rats. This protective effect is associated with changes of PI3K/AKT pathway and upregulation of IRAK-M, which is mediated by TLR4 signaling pathway. The specific mechanism is still unclear, but the phenomenon of endotoxin tolerance is identified as a state of generalized dampening of inflammatory pathways. A substantial body of researches shows that a low dose of LPS leads to a shift away of macrophages from a proinflammatory response toward an anti-inflammatory response [9].

The occurrence of uveitis is related to the local infiltration of neutrophils and the apoptosis of peripheral blood lymphocytes. Cell apoptosis may be conducive to the elimination of inflammation, while abnormal apoptosis may lead to prolonged inflammation and easy recurrence [10]. Macrophage colony-stimulating factor (M-CSF), also known as colony-stimulating factor-1 (CSF-1), is playing a significant role in regulating the differentiation of internal and external granulocytes or monocytes and promoting the maturation of granulocytes. It is considered as one of the important markers of nonspecific cellular immune response in the body's anti-inflammatory process [11]. Although macrophages and other mononuclear cell lines are also regulated by other growth factors, the most important regulator is CSF-1. Recent studies have shown that downregulated CSF-1 can block cell cycle and induce apoptosis [12]. It is not clear whether downregulated CSF-1 is involved in reducing uveitis, which this study intends to explore. Leucine-rich repeats (LRRs) are the main components of the extracellular domain of TLR4, among which leucine-rich repeat protein-1 (LRR-1) plays an important role in immune tolerance. Therefore, this study attempted to investigate whether CSF-1 and LRR-1 are involved in endotoxin tolerance in uveitis through the TLR4 pathway and their exact mechanisms. The results of this study provide a new idea for the targeted therapy of uveitis.

## 2. Materials and Methods

### 2.1. Animals and Reagents

Male SPF Wistar rats (8-10 weeks old, weighing 180-200 g) were purchased from the Vital River Laboratory Animal Technology Co., Ltd. (Beijing, China) and maintained in an air-conditioned room with 12 h light/12 h dark cycles. Food and water were unlimited. All experimental procedures were in accordance with the Institute for Laboratory Animal Research guidelines (Guide for the Care and Use of Laboratory Animals).

Lipopolysaccharide (V. cholerae, classical Biotype, serotype Ogawa) was provided by the Lanzhou Biological Product Research. Rabbit CSF-1 antibody was purchased from LifeSpan BioSciences, Inc., USA. Rabbit LRR-1 antibody was purchased from Wuhan Proteintech Group, Inc. Rabbit NF-*κ*B p65 antibody was purchased from Abcam Co., Ltd., UK. HRP-marked goat anti-rabbit IgG antibody and CY3-marked goat anti-rabbit fluorescence IgG antibody were purchased from Wuhan Servicebio technology, Co., Ltd. RevertAid First Strand cDNA Synthesis Kit was purchased from ThermoFisher Scientific Co., Ltd., USA. FastStart Universal SYBR Green Master(Rox) was purchased from F. Hoffmann-La Roche Ltd., Switzerland. Rat INF-*γ* ELISA kit, Rat IL-6 ELISA kit, and Rat IL-17 ELISA kit were purchased from Cusabio.

### 2.2. Animal Model and Experimental Groups

Endotoxin-induced uveitis (EIU) was induced by a single subcutaneous injection of 200 *μ*g LPS dissolved in 0.1 ml sterile saline (1 mg/kg) as previously described [13]. All animals were randomly divided into three groups: normal control (NC) group, endotoxin-induced uveitis (EIU) group, and endotoxin tolerance (ET) group. In the ET group, endotoxin tolerance was induced by daily subcutaneous injection of 0.1 mg/kg LPS for five days [14]. The other two groups of rats were treated with sterilized saline in the same manner as pretreatment. On day 6, the animals in the ET and EIU groups received a single subcutaneous injection of 200 *μ*g LPS to induce EIU. The NC group received subcutaneous injection of 0.1 ml sterile saline.

### 2.3. Clinical Evaluation

Rat eyes were examined under a slit lamp at 24 h after injection of 200 *μ*g LPS or sterile saline. The severity of the ocular inflammation was evaluated according to the scoring criteria (Table 1) [15].

### 2.4. Histopathological Examination

The rats were killed after clinical evaluation. Both eyes were enucleated and placed in 10% neutral buffered formalin solution for 24 hours. The eye specimens were dehydrated in a graded ethanol series and embedded in paraffin. Sagittal sections (4-5 *μ*m thick), cut near the optic nerve head, were stained with hematoxylin and eosin. As previously described [16], anterior chamber tissues were scored for severity of inflammation as follows: 0: normal tissue; 1: dilated iris vessels and thickened iris stroma with exudate, protein, and/or a few scattered inflammatory cells in the anterior chamber; 2: infiltration of inflammatory cells into the stroma of the iris and/or ciliary body, with a moderate number of inflammatory cells within the anterior chamber; 3: heavy infiltration of inflammatory cells within the iris stroma and ciliary body and a heavy infiltration of inflammatory cells within the anterior chamber; and 4: heavy exudation of cells in dense protein aggregation in the anterior chamber and inflammatory cell deposits on the corneal endothelium.

### 2.5. Real-Time PCR Analysis

Iris ciliary body tissues (ICB) were separated under a stereomicroscope and total RNA was extracted with Trizol reagent. Signal-stranded cDNA was synthesized according to the kit instructions. Transcription levels of CSF-1, LRR-1, and NF-*κ*B p65 were analyzed by real-time PCR performed in the SLAN fluorescence quantitative PCR detection system (Shanghai Hongshi Medical Equipment Co., Ltd.) with FastStart Universal SYBR Green Master (Rox). All samples were run in triplicate. The CT values of the genes of interest were first normalized with *β*-actin from the same sample, and *ΔΔ* CT method was used to quantify mRNA expression levels. The primer sequences used in the present study were listed in Table 2.

### 2.6. Western Blot

ICB were separated under a stereomicroscope. Rinsed with PBS for 3 times and lysed with RIPA extraction reagent to extract total proteins. Oscillated in the ice water for 30 minutes, centrifuged for 10 minutes, and then collected supernatant fluid. BCA method was used to measure protein concentration. SDS-PAGE electrophoresis, film transfer, and closure were performed. Incubated with anti-CSF-1, anti-LRR-1, and anti-NF-*κ*B p65 at 4°C for 3 hours, respectively. Rinsed with TBST at room temperature for 3 times. Incubated with HRP-marked goat anti-rabbit IgG secondary antibody at a dilution of 1 : 3000 for 30 minutes. Rinsed with TBST again. ECLA and ECLB were used for chemiluminescence. Detected and analyzed the gray values of protein strips. All values were normalized with *β*-actin as loading control. Each sample was collected from five rats (10 eyes).

### 2.7. Immunofluorescence Analysis

After antigen repair, ICB sections were rinsed with PBS and sealed with BSA for 30 minutes. Incubated with anti-CSF-1 (at a dilution of 1 : 50), anti-LRR-1 (at a dilution of 1 : 100), and anti-NF-*κ*B p65 (at a dilution of 1 : 500) at 4°C for the night, respectively. Rinsed with PBS for 3 times and incubated with the CY3-marked goat anti-rabbit fluorescence IgG antibody (at a dilution of 1 : 300) at dark room temperature for 50 minutes. Rinsed again and stained nuclear with DAPI. Incubated at dark room temperature for 10 minutes. Rinsed with PBS for 3 times and sealed the film. Observed and collected the image under a fluorescence microscope (Nikon Eclipse C1, Nikon, Japan). The images were captured, and the pairs of images were superimposed for colocalization analysis using image management software (Adobe Photoshop CS3. 10.0; Adobe Systems, Mountain View, CA).

### 2.8. The Concentrations of INF-*γ*, IL-6, and IL-17 in Aqueous Humor

Collected aqueous humor at 24 h after injection of 200 *μ*g LPS or sterile saline. Methods: anesthetized the rats and cleaned the conjunctival sac, the aqueous humor samples were collected from both eyes of all rats using a 30-gauge needle attached to a 1 ml syringe under a microscope [17]. The concentrations of INF-*γ*, IL-6, and IL-17 in the samples were determined by enzyme-linked immunosorbent assay.

### 2.9. Statistical Analysis

Quantitative data were analyzed with one-way analysis of variance (ANOVA) (SPSS 19.0; SPSS Inc., Chicago, IL). Values of *P* < 0.05 were considered statistically significant.

## 3. Results

### 3.1. The Inflammatory Reaction Was Significantly Reduced in the ET Group

**Figure 1:**
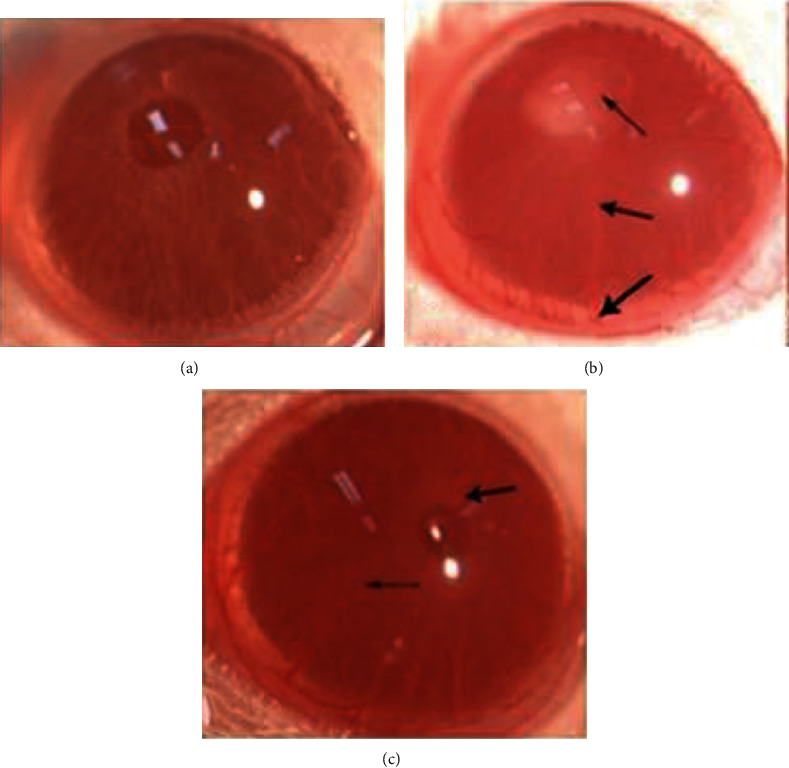
Clinical signs of anterior segment. (a) No signs of inflammation were observed in the NC group. (b) In the EIU group, we observed obvious closed pupil membrane (the top arrow), iris blood vessels dilation (the middle arrow), and purulent exudate (the bottom arrow). (c) The iris blood vessels dilation (the arrow on the left) and the pupil constriction (the arrow on the right) were slighter in the ET group; no other obvious inflammation was found.

**Figure 2:**
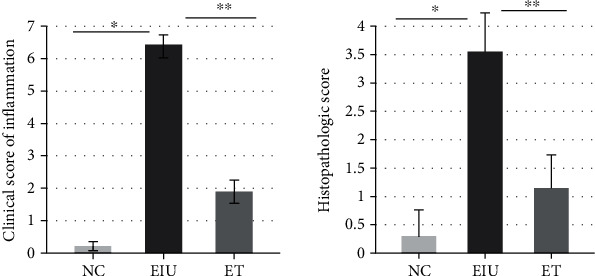
Clinical score results. All data shown as mean ± standard deviation (*n* = 20, eye). ∗∗*P* < 0.05, significantly different from EIU group.

Typical signs of anterior uveitis were observed 24 hours later in the EIU group with a slit lamp, including obvious iris blood vessels dilation, closed pupil membrane, and hypopyon (Figure 1(b)). While the inflammation in the ET group was mild, both iris blood vessels dilation and pupil constriction were slighter. Only a small amount of exudation was observed occasionally (Figure 1(c)). The NC group showed no signs of inflammation (Figure 1(a)). The score of the ET group was 1.90 ± 0.71, significantly lower than that of the EIU group 6.43 ± 0.50 (*F* = 979.446, *P* < 0.05) (Figure 2).

### 3.2. Inflammatory Cell Infiltration Was Obviously Reduced in the ET Group

**Figure 3:**
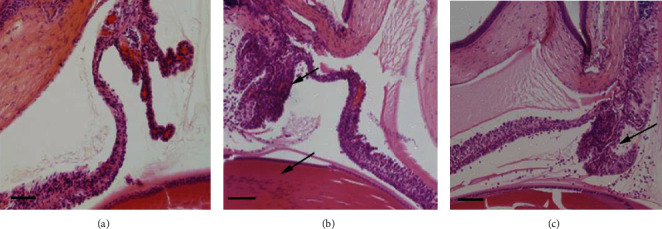
Histopathological findings. (a) No inflammatory cell infiltration was observed in the NC group. (b) In the EIU group, there was a large infiltration of inflammatory cells (the top arrow) in the iris stroma and corneal endothelium (the bottom arrow pointed to the lens). (c) Only a small amount of scattered inflammatory cells were observed in the ET group (the arrow pointed to the iris ciliary body). Scale bars: 100 *μ*m.

The results of HE stain were consistent with the clinical characteristic. A large number of inflammatory cells infiltrated into the iris stroma and corneal endothelium in the EIU group (Figure 3(b)). While the exudation in the ET group was obviously reduced (Figure 3(c)). There was almost no inflammatory cell infiltration in the NC group (Figure 3(a)). Histopathological score of the ET group was 1.15 ± 0.59, significantly lower than that of the EIU group 3.55 ± 0.69 (*F* = 164.393, *P* < 0.05) (Figure 2).

### 3.3. Low Dose of LPS Pretreatment Inhibited the Expression of CSF-1 and NF-*κ*B P65

**Figure 4:**
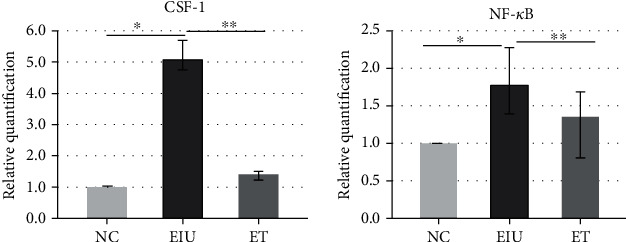
Real-time PCR of CSF-1 and NF-*κ*B P65 in ICB. The mRNA expression of CSF-1 and NF-*κ*B P65 in the EIU group were significantly higher than that in the NC group (∗*P* < 0.05) and ET group (CSF-1: *F* = 602.127, ∗∗*P* < 0.05; NF-*κ*B P65: F =24.173, ∗∗*P* < 0.05). All data shown as mean ± standard deviation from ten independent experiments.

**Figure 5:**
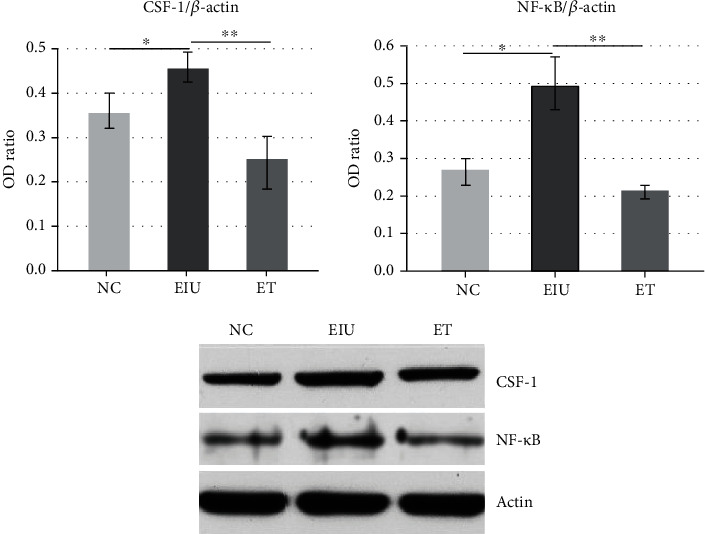
Western blot of CSF-1 and NF-*κ*B P65 in ICB. The protein expression of CSF-1 and NF-*κ*B P65 in the EIU group were significantly higher than that in the NC group (∗*P* < 0.05) and ET group (CSF-1: *F* = 31.081, ∗∗*P* < 0.05; NF-*κ*B P65: *F* = 53.861, ∗∗*P* < 0.05). All data shown as mean ± standard deviation from ten independent experiments.

As shown in Figure 4, the application of 200 *μ*g LPS upregulated the mRNA expression of CSF-1 and NF-*κ*B P65 (mean ± SD of the relative CSF-1 mRNA expression: 1.00 ± 0.04 in the NC group, 5.08 ± 0.09 in the EIU group, 1.40 ± 0.06 in the ET group; mean ± SD of the relative NF-*κ*B P65 mRNA expression: 1.00 ± 0.03 in the NC group, 1.78 ± 0.08 in the EIU group, 1.35 ± 0.06 in the ET group). Moreover, the mRNA expression of CSF-1 and NF-*κ*B P65 in the ET group were downregulated after pretreatment with low dose of LPS (3.63-fold and 1.32-fold, respectively). The results of the protein expression also showed the same trend (Figure 5). The results indicated that low dose of LPS pretreatment inhibited the expression of CSF-1 and NF-*κ*B P65.

### 3.4. Low Dose of LPS Pretreatment Promoted the Expression of LRR-1

**Figure 6:**
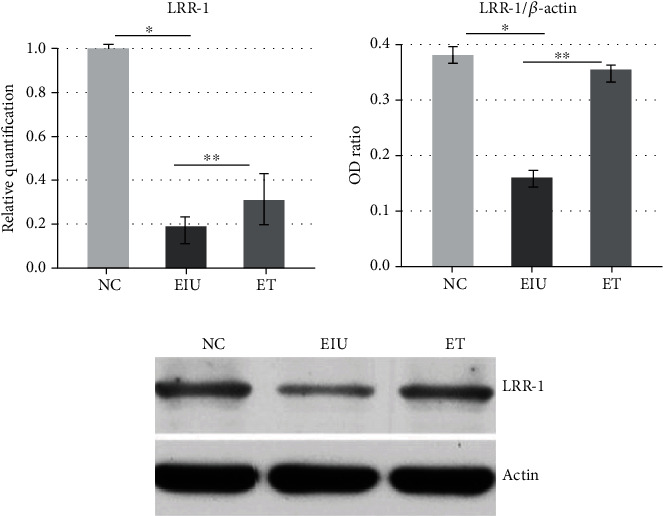
Real-time PCR and Western blot of LRR-1 in ICB. Both mRNA and protein expression of LRR-1 in the EIU group were lower than that in the NC group (∗*P* < 0.05) and ET group (the mRNA expression of LRR-1: *F* = 348.862, ∗∗*P* < 0.05; the protein expression of LRR-1: *F* = 388.500, ∗∗*P* < 0.05). All data shown as mean ± standard deviation from ten independent experiments.

Compared to the NC group, both mRNA and protein expression of LRR-1 declined dramatically in the EIU group. Though the mRNA and protein expression of LRR-1 in the ET group were still lower than those in the NC group, it is significantly higher than those in the EIU group (mean ± SD of the relative LRR-1 mRNA expression: 1.00 ± 0.02 in the NC group, 0.19 ± 0.03 in the EIU group, 0.31 ± 0.07 in the ET group, Figure 6). Low dose of LPS pretreatment induced a 1.63-fold increase in LRR-1 mRNA expression compared to the EIU group. The results indicated that low dose of LPS pretreatment promoted the expression of LRR-1.

### 3.5. Immunofluorescence

**Figure 7:**
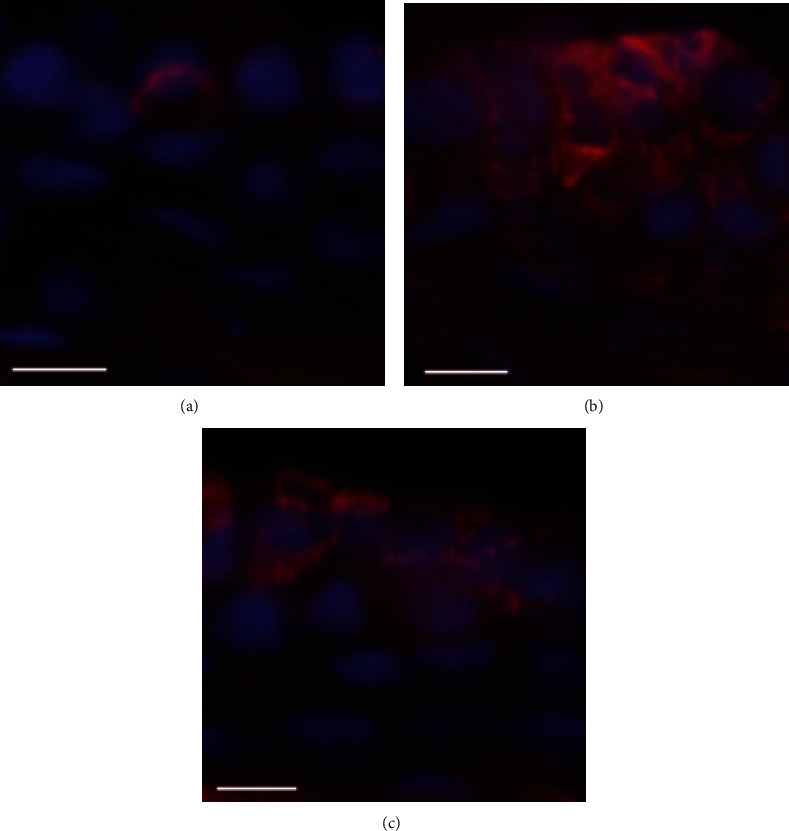
The result of immunofluorescence of CSF-1 in ICB. In the NC group, the positive expression of CSF-1 was few (a). In the EIU group, the positive expression of CSF-1 was widespread (b). In the ET group, the positive expression of CSF-1 was significantly reduced compared to the EIU group (c). Scale bars: 50 *μ*m.

**Figure 8:**
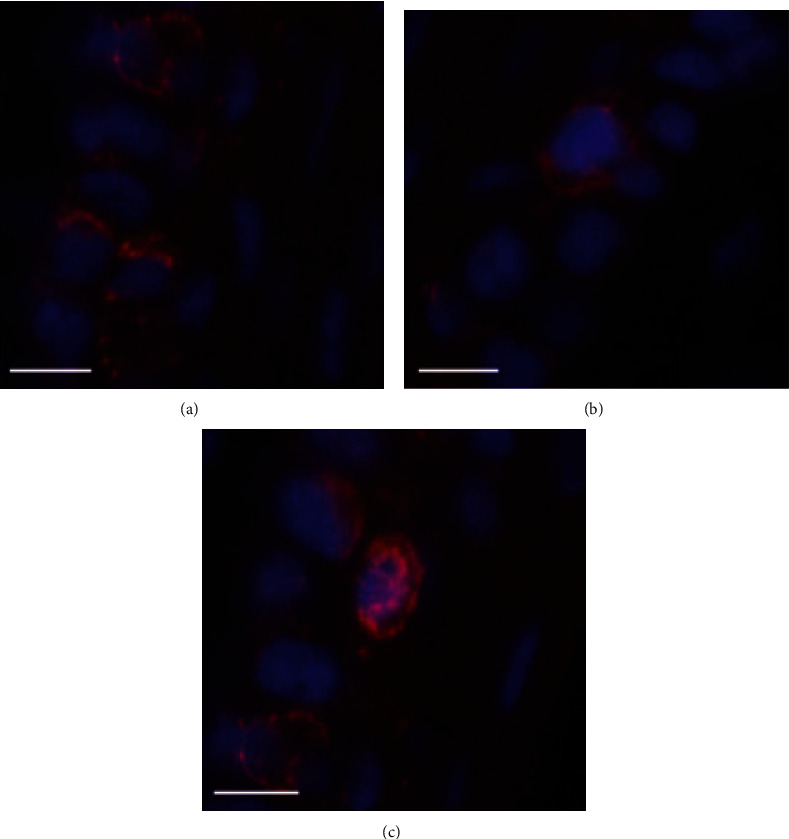
The result of immunofluorescence of LRR-1 in ICB. In the NC group, the positive expression of LRR-1 was clearly visible (a). In the EIU group, the positive expression of LRR-1 was occasionally observed (b). In the ET group, the positive expression of LRR-1 was higher than that in the EIU group, but still slightly lower than that in the NC group. Scale bars: 50 *μ*m.

The positive expression of CSF-1 was few in the absence of LPS stimulation (Figure 7(a)). After 200 *μ*g LPS stimulation, a large number of positive expression of CSF-1 was found (Figure 7(b)), which indicated that CSF-1 was activated. However, the positive expression of CSF-1 was significantly reduced in the low dose of LPS pretreatment group (Figure 7(c)), indicating that low dose of LPS pretreatment could inhibit the activation of CSF-1. Different from CSF-1, the positive expression of LRR-1 was clearly visible in the NC group (Figure 8(a)) but occasionally observed in the EIU group (Figure 8(b)), indicating that the activation of LRR-1 was inhibited after 200 *μ*g LPS stimulation. After low dose of LPS pretreatment, the positive expression of LRR-1 was higher than that in the EIU group (Figure 8(c)), but still slightly lower than that in the NC group, indicating that the inhibition of the LRR-1 activation could be partially relieved by low dose of LPS pretreatment. In other words, low dose of LPS pretreatment could promote the activation of LRR-1.

### 3.6. The Levels of INF-*γ*, IL-6, and IL-17 in Aqueous Humor

**Figure 9:**
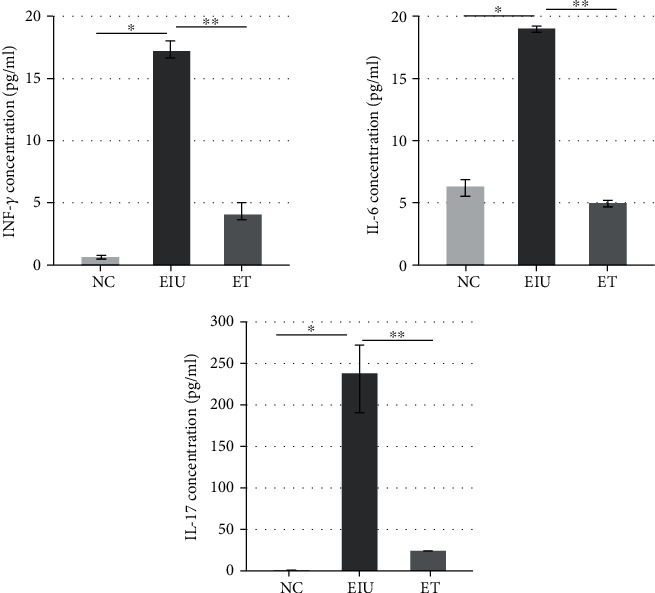
The levels of INF-*γ*, IL-6, and IL-17 in aqueous humor. INF-*γ*, IL-6, and IL-17 were significantly increased in the EIU group (∗*P* < 0.05) and significantly decreased in the ET group (INF-*γ*: *F* = 434.456, ∗∗*P* < 0.05; IL-6: *F* = 346.117, ∗∗*P* < 0.05; IL-17: *F* = 572.173, ∗∗*P* < 0.05). All data shown as mean ± standard deviation (*n* = 20).

As shown in Figure 9, the levels of INF-*γ* (17.17 ± 0.24 pg/ml), IL-6 (18.99 ± 0.11 pg/ml), and IL-17 (237.71 ± 1.64 pg/ml) in aqueous humor in the EIU group were significantly higher than that in the ET group, INF-*γ* (4.06 ± 0.32 pg/ml), IL-6 (4.98 ± 0.17 pg/ml), and IL-17 (23.83 ± 3.15 pg/ml).

## 4. Discussion

Endotoxin tolerance is a reduced responsiveness of cells like macrophages/monocytes to subsequent endotoxin challenge following a prior exposure to low dose of endotoxin [6]. Although endotoxin tolerance has been well studied in many diseases, little of it is known in uveitis.

In order to elucidate the potential mechanism of endotoxin tolerance in uveitis, this study established an endotoxin tolerance model for uveitis and detected the expression of IL-17, INF-*γ*, and IL-6 in rats' aqueous humor after clinical evaluation. Cytokines like IL-17, INF-*γ*, and IL-6 in aqueous humor are locally produced [15, 18]. All of them are proinflammatory cytokines that have been shown to be involved in several forms of infectious and noninfectious uveitis [18–20]. Chen et al. found that the levels of IL-17a, INF-*γ*, and IL-6 in aqueous humor were correlated with the activity of acute anterior uveitis [21]. Xu et al.'s research suggested that the levels of INF-*γ* and IL-10 were related to the severity of uveitis [22]. Huang et al.'s report was arguably the first to implicate IL-17F in the pathogenesis of acute anterior uveitis [23]. Drozdova et al. found a statistically significant increase in the levels of IL-17, IFN-*γ*, TNF-*α*, and IL-10 in all patients with uveitis as compared to the control group [24]. The last three cytokines have been shown to play an essential role in the mechanism of endotoxin tolerance [8, 25, 26]. Hoekzema et al. found that after a single injection of endotoxin, the levels of IL-6 in rats' aqueous humor significantly increased. Although repeated injection of endotoxin resulted in the systemic release of IL-6, no IL-6 was detected in aqueous humor and no uveitis was found [15]. Our results showed that rats in the EIU group reacted with a strong local production of cytokines like IL-17, INF-*γ*, and IL-6 in aqueous humor, while rats in the ET group showed only moderate expression of these three cytokines. The results were consistent with previous reports. The decrease in the expression of these cytokines could explain the decreased ocular inflammation in rats in the ET group. The anti-inflammatory effect of low dose of LPS pretreatment was confirmed again. Furthermore, the results indicated that the reduction of local proinflammatory cytokines was closely involved in the protective mechanism of endotoxin tolerance in uveitis. It is well known that the MyD88-dependent TLR4 signaling pathway is the most likely pathogenesis of EIU. TLR4 activates inflammation through NF-*κ*B p65 pathway. When the body or cells are stimulated by LPS, a series of reactions can promote the activation of NF-*κ*B p65, and the activation of NF-*κ*B p65 can induce the synthesis of proinflammatory cytokines such as IL-17, INF-*γ*, and IL-6, eventually resulting in inflammation [5]. During this process, MAPK and P13K/AKT are important factors in TLR4-MyD88-NF*κ*B p65 signaling pathway. In particular, the P13K/AKT signaling pathway has been proved to be associated with the protective effect of low dose of LPS pretreatment on EIU in rats [8]. Studies have shown that CSF-1 can trigger various signal transduction pathways such as MAPK and P13K/AKT, and thus play its biological role through the target cell membrane specific receptor [27]. So we speculate that there may be a correlation between CSF-1 and TLR4-NF*κ*B p65, through which can regulate ocular inflammation. Therefore, the next step is to investigate whether CSF-1 is involved in endotoxin tolerance in uveitis and its relationship with TLR4 and NF-*κ*B p65.

CSF-1, produced by activated macrophages, lymphocytes, tumor cells, and other cells, can initiate and enhance the killing effect of macrophages. It is one of the most common proinflammatory factors in a variety of inflammatory diseases [28, 29]. The increased expression of CSF-1 can effectively promote inflammatory response. Consistent with these observations, our results showed that the levels of CSF-1 in the EIU group were significantly higher than that in the ET group and the NC group. Clinical signs, histopathology, and cytokines in aqueous humor also suggested severe inflammation. However, this study mainly studied the levels of CSF-1 in the iris ciliary body tissues, which was not found in previous studies. Recent studies have shown that downregulated CSF-1 induces apoptosis [12]. Apoptosis has been reported to be beneficial for the elimination of inflammation [10]. Thus, we assumed that downregulated CSF-1 was involved in alleviating EIU. Our study showed much lower expression of CSF-1 at both mRNA and protein levels in the low dose of LPS pretreatment group than that in the EIU group. Correspondingly, ocular inflammation was reduced in the ET group, as demonstrated by anterior signs and histopathology. Immunofluorescence also showed an inhibition of the CSF-1 activation. These results were concordant with our hypothesis as well as the literature. Furthermore, the expression of NF-*κ*B p65 and CSF-1 were inhibited simultaneously, maybe there are two reasons for this: first, they may be regulated by the same signaling pathway; second, they may even have an upstream and downstream relationship with each other. As mentioned above, the activation of NF-*κ*B p65 mediated by TLR4 is a classical pathway in EIU animal models [5], so CSF-1 may also be regulated by the TLR4 signaling pathway. Zhang et al. reported that CSF-1 can promote the expression of TNF-*α* through the NF-*κ*B pathway and aggravate the inflammatory response [30], further demonstrating the positive correlation between CSF-1 and NF-*κ*B p65. In summary, the downregulation of CSF-1 induces endotoxin tolerance. Reducing the levels of CSF-1 and NF-*κ*B P65 through TLR4 pathway and then leading to a reduction of proinflammatory cytokines appear to be a possible mechanism. Further experiments such as using CSF-1 deficient/overexpressed animals or using CSF-1 receptor antagonist in the experimental uveitis are needed to confirm whether CSF-1 is an effective target for modulating uveitis.

In addition, we also studied the expression of LRR-1, one of the main components of the extracellular domain of TLR4 [31]. Up to now, though 375 LRR proteins have been identified, the function of most of them is still unknown [32]. LRR-1 is one of them. Jang et al. found that overexpression of LRR-1 inhibited the activation of NF-*κ*B [33]. In the present study, we found similar results; both the gene and protein expression of LRR-1 were significantly increased in the low dose of LPS pretreatment group, and the immunofluorescence results also showed an activation of LRR-1, while the expression of NF-*κ*B P65 was inhibited. The results suggested that the upregulation of LRR-1 was involved in endotoxin tolerance in uveitis. LRR-1 has been shown to negatively regulate the signaling pathway [33]. However, the current research on LRR-1 is limited, and the specific mechanism of its involvement in endotoxin tolerance is worthy of further study.

In conclusion, low dose of LPS pretreatment has a protective effect on endotoxin-induced uveitis in rats. This protection is related to upregulation of LRR-1 and downregulation of CSF-1, which may be mediated by TLR4 signaling pathway. CSF-1 is expected to be a new therapeutic target for uveitis.

## Figures and Tables

**Table 1:** Scoring system for clinical evaluation of uveitis.

Clinical signs	Grade of uveitis (score)
Iris hyperemia	
Absent	0
Mild	1
Moderate	2
Severe	3
Pupil	
Normal	0
Miosed	1
Exudate in anterior chamber	
Absent	0
Small	1
Large	2
Hypopyon	
Absent	0
Present	1
Maximum possible score	7

**Table 2:** Primer sequences.

Primer	Sequences(5′-3′)
CSF-1	ACTATAAGGAACAGAACGAGGCC
	TGAGAATCATCCCAAGCCAAGT
LRR-1	TCAGCAGCTCGAAACAAACTTAG
	CAAATCTTGGCAAAGGTGGTAA
NF-*κ*B p65	AGAACAGCAAGGCAGCACTCC
	AGGTGTCGTCCCATCGTAGGT
*β*-Actin	TGCTATGTTGCCCTAGACTTCG
	GTTGGCATAGAGGTCTTTACGG

## Data Availability

The data used to support the findings of this study are included within the article.
